# What is the relevance of quality of life assessment for patients with attention impairment?

**DOI:** 10.1186/1477-7525-11-70

**Published:** 2013-04-25

**Authors:** Karine Baumstarck, Mohamed Boucekine, Irina Klemina, Françoise Reuter, Valérie Aghababian, Anderson Loundou, Jean Pelletier, Pascal Auquier

**Affiliations:** 1 Self-perceived Health Assessment Research Unit, School of Medicine, Université de la Méditerranée, Marseille, France; 2 Departments of Neurology and CRMBM CNRS6612, Timone University Hospital, APHM, Marseille, France; 3 EA 3273 Psychology of Cognition, Language, and Emotion Research Centre, Aix-Marseille University, Marseille, France

**Keywords:** Attention dysfunction, Multiple sclerosis, Quality of life, Validity, Reliability, MusiQoL

## Abstract

**Background:**

Attention disturbances are frequently observed in multiple sclerosis (MS) patients. The aim of this study was to provide new evidence regarding the suitability of using self-reported QoL information in this impaired population by exploring the construct validity, reliability, and external validity of a MS-specific quality of life (QoL) instrument.

**Methods:**

*Design:* cross-sectional study. *Inclusion criteria*: MS patients of any disease subtype. *Data collection*: sociodemographic (age, gender, marital status, education level, and occupational activity) and clinical data (Expanded Disability Status Scale, disease duration); QoL (MusiQoL and SF36); and attention performance (Wechsler Memory Scale and PASAT). According to the French norms, non-impaired and impaired populations were defined. For each population, suitability indices were provided to quantify how the structures matched with the initial structure of the reference population assessed in the validation study.

**Findings:**

One hundred and twenty-four consecutive patients were enrolled. The factor analysis performed in the impaired populations showed that the questionnaire structure adequately matched the initial structure of the MusiQoL. The unidimensionality of the dimensions was preserved, and the internal/external validity indices were close to those of the reference population.

**Conclusions:**

Our study suggests that attention impairment dysfunction did not compromise the reliability and validity of the self-reported QoL questionnaires.

## Introduction

The use of self-reported outcomes in subjects with cognitive dysfunction is of particular concern [1]. The main argument against using self-reported quality of life (QoL) information from patients with cognitive dysfunction is that QoL instruments were not developed for use with cognitively impaired individuals. Some authors argue that cognitively impaired individuals suffering from multiple sclerosis (MS) are unable to produce valid QoL measures [2,3], whereas other studies have suggested that cognitive decline in these patients does not compromise the reliability/validity of self-reported health measures [4-7]. In these previous studies, the assessment of cognitive function focused on aspects of memory [5,7] and executive functions [6].

Attention plays a role in cognitive processing and learning. Attention as a basic cognitive function represents an essential part of conscious perception and higher order cognitive functions and plays an important role in normal functioning. Attention is a broad term that is used to designate the processes that mediate the appropriate allocation of cognitive resources to relevant stimuli, such as objects, locations or moments. The limitation of this capacity may result from deficits in the automatic or controlled processing of information, as well as the incapacity to store and manipulate temporary information, the function known as working memory. In MS patients, cognitive performance is globally affected and impacts divided attention, focused attention, sustained attention, attentional flexibility, and response speed [8,9]. Despite the high occurrence of working memory and attention disturbances in MS patients [10,11], even during the early stages of the disease [12], no study has specifically examined the extent to which MS patients with attention dysfunction can validly self-report their QoL. Herein, we provide new evidence regarding the suitability of using self-reported QoL information in this impaired population. The aim of this study was to explore the construct validity, reliability and external validity of a MS-specific QoL instrument in MS subjects with or without attention impairment.

## Methodology

This study relied on a cross-sectional design and was performed in the neurology department of a public French academic teaching hospital (Marseille, France). The inclusion criteria were as follows: a MS diagnosis according to the McDonald criteria [13], age ≥ 18 years, any subtype of MS, outpatient status, no neurological disease (other than MS), no history of severe mental illness (except depressive disorder), no dementia (Mini Mental State Examination score < 24), no history of alcohol/drug abuse, and native French-speaking. The French Ethics Committee approved the study (Comité Consultatif de Protection des Personnes dans la Recherche Biomédicale, Marseille 2, France) and patients gave their written consent to participate. Sociodemographic (age, gender, marital status, education level, and occupational activity) and clinical (MS subtype and disease duration) data were recorded for each patient. MS disability was assessed using the Expanded Disability Status Scale (EDSS).

QoL was assessed using the French version of the Multiple Sclerosis International Quality of Life questionnaire (MusiQoL) and the Short Form 36 (SF36). The MusiQoL is a well-validated MS-specific questionnaire that describes the nine dimensions and yields a global index score: activity of daily living (ADL), psychological well-being (PWB), symptoms (SPT), relationships with friends (RFr), relationships with family (RFa), relationships with health care system (RHCS), sentimental and sexual life (SSL), coping (COP), and rejection (REJ). The SF36 is a generic questionnaire [14] describing eight subscales: physical function (PF), social function (SF), role physical (RP), role emotional (RE), mental health (MH), vitality (V), bodily pain (BP), and general health (GH). Two composite scores (physical and mental, PCS-SF36 and MCS-SF36) were also calculated.

Attention performance was measured using two tests: the French version of the Paced Auditory Serial Addition Test (PASAT) [15] and the attention/concentration subscale of the French version of the Wechsler Memory Scale (attention_WMS) [16]. The attention_WMS score was calculated using 3 subtests (mental control, digit span, and visual memory). By comparing with French normative values according to age group [16], a patient was considered cognitively impaired if their score was more than 1 standard deviation (SD) below the normative value. The PASAT score was generated using the PASAT-3 s version of the test (number of correct responses). By comparing with French normative values according to sex, age, and educational level [17], a patient was considered to be cognitively impaired if their PASAT score was more than 1 SD below the normative value.

### Statistical analysis

Statistical analyses were performed on the populations defined above using the same procedure reported in the initial validation publication (reference population) [18]. For each group, psychometric properties were compared to those reported from the reference population. The structures of the MusiQoL were explored in the non-impaired and impaired populations using principal component factor analyses with varimax rotation [19,20] to determine how these structures matched with the initial structure of the MusiQoL. For each population, the proportion of factors identified from the initial nine-factor structure of MusiQoL and the proportion of items projected to their initial dimension were retrieved.

The multidimensional structure (construct validity) was verified using the multi-trait/multi-item analysis program [21]. Internal structural validity was assessed by calculating item-dimension correlations. Item internal consistency (IIC) was calculated by correlating each item with its scale, and item discriminant validity (IDV) was assessed by determining the extent to which items correlated with the dimension they were hypothesized to represent compared to correlations with other dimensions. Floor and ceiling effects were determined to assess the distribution of the responses. For each dimension, internal consistency reliability was evaluated using Cronbach’s alpha coefficient [22].

The unidimensionality of each dimension was calculated by computing item goodness-of-fit statistics (INFIT) issued from Rasch analyses [23]. INFIT values ranging from 0.7 to 1.2 ensure that all scale items tend to measure the same concept.

To assess external validity, Spearman’s correlation coefficients were used to determine relationships between the MusiQoL and SF36 dimensions in each group, and the associations between MusiQoL dimension scores and sociodemographic and clinical features were reported as in the validation study. For qualitative variables, the mean dimension scores of the MusiQoL were compared across patient groups (e.g., gender, educational level, marital status, and occupational status) using one-way analysis of variance. Quantitative variables (e.g., age, EDSS score, and MS duration) were analyzed using Spearman’s correlation coefficients. Acceptability was assessed by calculating the percentage of missing data per dimension. Data analyses were performed using SPSS 11.0, MAP-R, LISREL and WINSTEP software.

To quantify how each of the 4 structures matched with the initial structure (reference structure), suitability indices were calculated in accordance with a previous study [7]. Decision rules were established by experts in QoL and used to define satisfactory properties according to appropriate standards [19,20]. The means of different proportions were calculated to produce the suitability index of the ‘construct validity’ and the suitability index of the ‘external validity’.

## Results

A total of 124 patients participated in the study. The mean age was 45 years (SD 11), 71 (57,3%) of the patients were women, and 58 (47,2%) had more than 12 years of education. MS subtypes included 61 (49,2%) secondary progressive, 36 (29,0%) relapsing-remitting, 20 (16,1%) primary progressive, and 7 (5,6%) clinically isolated syndromes. The EDSS median was 4.75 (minimum 1.00, maximum 8.00), and the median disease duration was 9.86 years (minimum 0, maximum 31). After comparison to the French normative values, 75 non-impaired and 39 impaired individuals were identified based on the attention_WMS, and 32 non-impaired and 73 impaired individuals were identified based on the PASAT.

### Construct validity

The 9-factor structure of the MusiQoL was clearly detected in the PASAT-impaired population, and 8 factors were detected in the attention_WMS-impaired population (the REJ dimension was not detected). The percentage of items well-projected to their initial dimension was higher in the impaired populations (range: 80,6 to 100%) compared to the non-impaired populations (Additional files 1 and 2). The proportion of dimensions with IIC and the proportion of dimensions with IDV that were greater than 0.2 from the reference dimension were moderately satisfactory. In contrast, the correlation of each item with its associated dimension was higher than with the others in 6 dimensions in the PASAT-impaired population and 7 dimensions in the attention_WMS-impaired populations. Cronbach’s alpha coefficients were satisfactory in at least 6 of 9 dimensions in the two impaired populations. The INFIT statistics were satisfactory in the attention_WMS-impaired population, but less satisfactory for the PASAT-impaired group. The proportions of dimensions with floor and ceiling effects less than 10% from the reference are described in Table 1. Additional details are provided in Table 2. The suitability indices of construct validity in the impaired populations were populations were detailed in Table 1.

**Table 1:** Suitability indices of construct validity and external validity according to the attention status

	**Attention_WMS**		**PASAT**	
**Construct validity**	**NI 75**	**I 39**	**NI 32**	**I 73**
% of identified factors	88,9	88,9	88,9	100
% of well-projected items	57,1	80,6	83,9	100
% of dimensions with IIC non exceeded 0,2 from ref	44,4	77,8	77,8	44,4
% of dimensions with IDV non exceeded 0,2 from ref	33,3	22,2	0	44,4
% of dimensions with IDV < IIC	88,9	77,8	77,8	66,7
% of dimensions with Cronbach’s alpha coefficients > = 0,7 or > = ref	77,8	66,7	77,8	77,8
% of dimensions with INFIT ranged [0,7-1,3]	83,3	83,3	66,7	44,4
% of dimensions with MV < 10% from ref	100	88,9	88,9	88,9
% of dimensions with Floor < 10% from ref	77,8	33,3	77,8	55,6
% of dimensions with Ceiling < 10% from ref	66,7	77,8	66,7	66,7
**Total**	71,8	69,7	70,6	68,9
**External validity**	**NI 75**	**I 39**	**NI 32**	**I 73**
SF36: % of dimensions meeting three conditions*	88,9	88,9	55,6	88,9
Age: % of dimensions with correlation coefficient < 0,40	100	100	100	100
EDSS: % of dimensions meeting two conditions**	100	88,9	88,9	88,9
MS duration: % of dimensions with correlation coefficient < 0,40	100	100	100	100
Gender: % of dimensions with ES < 0,2 from ref	88,9	100	100	100
Educational level: % of dimensions with ES < 0,2 from ref	55,6	44,4	66,7	44,4
Marital status: % of dimensions with ES < 0,2 from ref	100	88,9	77,8	100
Occupational status: % of dimensions with ES < 0,2 from ref	55,6	55,6	55,6	44,4
**Total**	86,1	83,3	80,6	83,3

* MusiQoL and SF36: the three conditions were: i) correlation coefficient between ADL and PF or RP or V higher than 0,50 and stronger than the other correlations; ii) correlation coefficient between PWB and MH higher than 0,50 and stronger than the other correlations; iii) all other correlation coefficients inferior to 0,40.

** MusiQoL and EDSS: the two conditions were: i) correlation coefficient between ADL and EDSS > 0,4 and stronger than the other correlations; ii) all other correlation coefficients inferior to 0,40, The score was 100% when all the dimensions met the condition.

*NI* non impaired, *I* impaired, Ref reference population.

**Table 2:** Internal structural validity/reliability/unidimensionality according to the cognitive status

	**1. attention_WMS**													
	**IIC**^**1 **^**Min-Max**		**IDV**^**2 **^**Min-Max**		**Floor %**		**Ceiling %**		**Alpha**^**3**^		**INFIT**^**4**^		**MV**^**5 **^**%**	
	**NI 75**	**I 39**	**NI 75**	**I 39**	**NI 75**	**I 39**	**NI 75**	**I 39**	**NI 75**	**I 39**	**NI 75**	**I 39**	**NI 75**	**I 39**
ADL	0,47-0,76	**0,12**-0,74	0,02-0,43	0,01-**0,43**	1,5	2,7	0	0	0,85	0,81	0,75-**1,49**	**0,44-2,29**	3,7	3,8
PWB	0,54-0,81	**0,62**-0,84	0,00-0,49	0,04-**0,58**	1,5	2,7	1,5	0	0,84	0,84	**0,64**-1,26	0,75-1,2	3,7	3,2
RFr	0,75-0,82	0,61-0,81	0,01-0,43	0,00-0,33	1,5	2,7	10,4	5,4	0,89	0,83	0,74-1,17	**0,51**-1,23	3,1	2,6
SPT	0,47-0,65	0,21-0,51	0,02-0,28	0,01-0,34	0	0	4,5	2,7	0,76	**0,61**	0,75-1,12	0,85-1,28	3,3	2,6
RFa	0,54-0,62	0,60-0,78	0,01-0,51	0,04-0,42	0	0	16,4	29,7	0,74	0,84	0,98-1,02	0,71-1,29	2,7	2,6
RHCS	0,49-0,65	0,41-0,59	0,01-0,28	0,01-0,45	0	0	13,4	5,4	0,74	**0,67**	0,75-**1,31**	0,8-1,08	3,1	2,6
SSL	0,53-0,53	0,77-0,77	0,02-0,43	0,02-0,26	20,9	18,9	9,0	10,8	**0,69**	0,87	0,97-1	0,99-1,03	11,3	5,1
COP	0,42-0,42	0,51-0,51	0,02-0,49	0,01-0,43	4,5	13,5	10,4	2,7	**0,59**	**0,67**	0,99-0,99	0,95-0,98	2,7	2,6
REJ	0,81-0,81	0,79-0,79	0,00-0,49	0,00-0,61	7,5	10,8	31,3	37,8	0,89	0,88	1,01-1,01	0,93-0,95	2,7	2,6
	**2. PASAT-3 s**													
	**IIC**^**1 **^**Min-Max**		**IDV**^**2 **^**Min-Max**		**Floor %**		**Ceiling %**		**Alpha**^**3**^		**INFIT**^**4**^		**MV**^**5 **^**%**	
	**NI 32**	**I 73**	**NI 32**	**I 73**	**NI 32**	**I 73**	**NI 32**	**I 73**	**NI 32**	**I 73**	**NI 32**	**I 73**	**NI 32**	**I 73**
ADL	0,36-0,70	0,43-0,63	−0,45-0,50	−0,24-0,31	31,8	33,7	12,1	5,3	0,86	0,83	0,72-1,85	0,72-1,42	0	1,4
PWB	0,62-0,83	0,63-0,81	−0,26-0,57	0,03-0,53	6,3	13,4	13,3	15,1	0,88	0,86	0,77-1,31	0,62-1,25	0	1,4
RFr	0,56-0,76	0,35-0,63	−0,43-0,37	−0,22-0,39	4,2	3,2	20,8	16,4	0,84	0,89	0,59-1,32	0,98-1,33	0	1,4
SPT	0,54-0,59	0,74-0,88	−0,24-0,59	−0,04-0,43	7,1	11,7	21,1	16,4	0,76	0,70	0,85-1,11	0,69-1,28	0	1,4
RFa	0,59-0,60	0,52-0,64	−0,40-0,38	−0,19-0,46	2,1	4,1	41,7	37,0	0,82	0,78	0,90-1,02	0,84-1,26	0	1,4
RHCS	0,59-0,63	0,42-0,61	−0,41-0,21	−0,03-0,25	2,1	4,1	25,0	25,1	0,81	0,71	0,83-1,03	0,83-1,31	0	1,4
SSL	0,49	0,68	−0,34-0,40	−0,09-0,38	17,2	27,4	26,6	15,8	0,68	0,79	0,90-1,13	0,96-1,03	6,3	5,5
COP	0,50	0,47	−0,36-0,41	−0,08-0,48	18,8	13	31,3	20,5	0,67	0,64	0,95-1,03	0,99	0	1,4
REJ	0,74	0,82	−0,34-0,47	−0,05-0,53	6,3	9,6	31,3	44,5	0,85	0,92	0,94-0,96	0,97	0	1,4

*ADL* activity of daily living, *PWB* psychological well-being, *RFr* relationships with friends, *SPT* symptoms, *RFa* relationships with family, *RHCS* relationships with health care system, *SSL* sentimental and sexual life, *COP* coping, *REJ* rejection.

*NI* non-impaired, *I* impaired.

^1^ Item-Internal Consistency, ^2^ Item Discriminant Validity, ^3^ Cronbach’s alpha, ^4^ Rasch statistics, ^5^ Missing Values.

Bold values: unsatisfactory values.

### External validity

Eight dimensions satisfied the 3 conditions defining the suitability of relationships between MusiQoL and SF36 scores, both for the PASAT- and attention_WMS-impaired populations (Tables 3 and 4). The suitability indices of age, disease duration, EDSS, gender, and marital status were satisfactory. Associations between the MusiQoL scores and educational level and occupational status were less satisfactory (Additional files 3 and 4). In summary, the suitability indices of external validity in the non-impaired populations were 83% (Table 1).

**Table 3:** Correlations between MusiQoL and SF36 scores according to the cognitive status based on Wechsler Memory Scale

**SF36**		**ADL**	**PWB**	**RFr**	**SPT**	**RFa**	**RHCS**	**SSL**	**COP**	**REJ**	**index**
Physical functioning	NI	0,721**	0,161	−0,071	−0,028	0,022	0,223	0,219	0,130	0,124	0,267*
	I	0,512**	0,332	−0,157	0,166	−0,202	0,041	0,206	0,261	0,150	0,303
Social functioning	NI	0,353**	0,211	0,149	0,405**	0,136	−0,085	0,222	0,398**	0,156	0,361**
	I	0,294	0,351*	0,004	0,052	0,173	0,456**	0,258	0,220	0,333	0,524**
Role physical	NI	0,564**	0,215	0,027	0,245*	−0,147	0,015	0,022	0,160	0,266*	0,233
	I	0,061	−0,066	0,029	−0,047	−0,083	0,075	−0,216	0,091	0,202	0,014
Role emotional	NI	0,285*	0,372**	0,051	0,444**	0,027	−0,212	0,284*	0,085	0,096	0,336**
	I	0,211	0,245	0,164	0,206	0,001	0,052	0,118	0,376*	0,266	0,395*
Mental health	NI	0,277*	0,728**	0,090	0,249*	0,056	0,037	0,192	0,437**	0,221	0,514**
	I	0,296	0,662**	0,097	0,178	0,306	0,120	0,380*	0,403*	0,320	0,686**
Vitality	NI	0,609**	0,319**	−0,129	0,379**	−0,165	0,108	0,063	0,132	0,117	0,222
	I	0,483**	0,299	−0,099	0,207	−0,089	0,207	0,257	0,103	0,156	0,353*
Bodily pain	NI	0,389**	0,286*	−0,034	0,242*	−0,244*	−0,003	0,062	0,089	0,136	0,218
	I	0,262	0,405*	−0,119	0,406*	0,031	−0,028	0,029	−0,010	0,279	0,313
General health	NI	0,482**	0,233	0,011	0,266*	0,181	−0,030	0,242	0,259*	0,082	0,309*
	I	0,564**	0,309	−0,012	0,127	−0,317	0,128	0,283	0,221	0,050	0,286
MCS	NI	0,258*	0,620**	0,090	0,486**	0,057	−0,127	0,228	0,372**	0,171	0,454**
	I	0,301	0,547**	0,187	0,180	0,261	0,254	0,375*	0,440**	0,379*	0,721**
PCS	NI	0,721**	0,034	−0,028	0,149	−0,105	0,153	0,081	0,090	0,144	0,165
	I	0,495**	0,161	−0,234	0,216	−0,404*	−0,041	−0,021	−0,002	0,105	0,049

*ADL* activity of daily living, *PWB* psychological well-being, *RFr* relationships with friends, *SPT* symptoms, *RFa* relationships with family, *RHCS* relationships with health care system, *SSL* sentimental and sexual life, *COP* coping, *REJ* rejection.

*MCS* mental composite score, *PCS* physical composite score.

*NI* non-impaired, *I* impaired.

Spearman rank correlation coefficients were presented.

Bold values: p < 0.05, *p-value <0.05, **p-value <0.01.

**Table 4:** Correlations between MusiQoL and SF36 scores according to the cognitive status based on PASAT

**SF36**		**ADL**	**PWB**	**RFr**	**SPT**	**RFa**	**RHCS**	**SSL**	**COP**	**REJ**	**index**
Physical functioning	NI	0,511**	−0,069	−0,047	−0,206	0,096	0,288	0,454*	0,113	0,215	0,264
	I	0,518**	0,160	−0,170	0,020	−0,023	0,121	0,049	0,097	−0,152	0,104
Social functioning	NI	0,377*	0,211	−0,088	0,404*	−0,128	−0,034	0,155	0,403*	−0,047	0,289
	I	0,325**	0,329**	0,087	0,219	0,158	0,080	0,154	0,326**	0,321**	0,404**
Role physical	NI	0,481**	0,072	−0,411*	0,092	−0,324	−0,143	0,124	0,100	0,166	0,005
	I	0,398**	0,153	0,073	0,162	−0,079	0,195	−0,097	0,212	0,212	0,222
Role emotional	NI	0,289	0,470*	−0,343	0,306	−0,171	−0,389*	0,227	0,159	0,185	0,224
	I	0,237	0,369**	0,262*	0,365**	0,200	−0,035	0,284*	0,155	0,196	0,438**
Mental health	NI	0,260	0,734**	−0,163	0,480**	−0,023	−0,118	0,015	0,401*	−0,099	0,433*
	I	0,304*	0,709**	0,216	0,184	0,296*	0,215	0,348**	0,343**	0,281*	0,632**
Vitality	NI	0,621**	0,353	−0,236	0,548**	−0,187	0,178	0,127	0,023	0,033	0,356
	I	0,540**	0,216	−0,104	0,086	−0,236	0,248*	0,069	0,191	0,024	0,188
Bodily pain	NI	0,483**	0,351	−0,161	0,390*	−0,318	−0,159	0,221	0,248	0,302	0,351
	I	0,348**	0,261*	−0,124	0,296*	−0,165	0,042	−0,032	−0,057	−0,006	0,143
General health	NI	0,602**	0,149	−0,107	0,245	0,108	0,122	0,290	0,096	−0,032	0,239
	I	0,449**	0,216	−0,128	0,226	−0,038	0,121	0,157	0,318**	−0,023	0,245
MCS	NI	0,164	0,645**	−0,288	0,528**	−0,059	−0,202	0,034	0,216	−0,132	0,251
	I	0,328**	0,615**	0,295*	0,320*	0,229	0,093	0,324*	0,423**	0,388**	0,637**
PCS	NI	0,679**	−0,192	−0,154	−0,044	−0,139	0,197	0,336	0,107	0,227	0,208
	I	0,542**	0,018	−0,281*	0,191	−0,324**	0,063	−0,131	0,006	−0,161	−0,055

*ADL* activity of daily living, *PWB* psychological well-being, *RFr* relationships with friends, *SPT* symptoms, *RFa* relationships with family, *RHCS* relationships with health care system, *SSL* sentimental and sexual life, *COP* coping, *REJ* rejection.

*MCS* mental composite score, *PCS* physical composite score.

*NI* non-impaired, *I* impaired.

Spearman rank correlation coefficients were presented.

Bold values: p < 0.05, *p-value <0.05, **p-value <0.01

## Discussion/conclusion

As reported in previous studies [4,6,7,24], our results support the conclusion that cognitively impaired MS patients, as defined by attention dysfunction, reliably and consistently answer the MusiQoL questionnaire. The suitability indices of the impaired populations were close to those of the non-impaired populations, and they could be considered completely acceptable considering the small sample size of some of the defined populations. The assessment of QoL in MS patients could be more widely used without concern over the adequacy of this approach for cognitively impaired patients.

Some strengths and limitations of the current study should be considered:

i) Considering only one type of dysfunction may not have accurately reflected global cognitive functioning. It has been well documented that it is unusual to observe one deficit in isolation in clinical practice. However, there is little consensus on the definition of ‘global’ cognitive dysfunction [25]. Despite this limitation, the findings of the present study support the relevance of self-reported quality of life assessments for patients with cognitive disorders. Future studies should provide similar explorations in MS populations with cognitive dysfunction defined with other definitions integrating combination of different composites.

ii) We did not consider factors previously associated to cognitive performance, such as depression [26], fatigue [27], and MS medications [28]. However, the aim of this study was to provide evidence supporting the conclusion that cognitively impaired MS patients reliably answer a self-reported QoL questionnaire regardless of the presence or absence of other factors that could have influenced their performance.

iii) The suitability indices used to define satisfactory properties relied on debatable decision rules and non-standardized calculation methods. Analyses of sensitivity should be performed.

iv) Replication of these findings in larger groups of patients is required.

v) Similar studies should be performed in other populations suffering from cognitive impairment, such as severe mental illness and elderly individuals, to determine whether these findings are specific to patients with MS.

## Competing interests

The authors declare that they have no competing interests.

## Authors’ contributions

Conception and design: PA, JP. Study coordination: PA, JP, KB. Inclusion and clinical data collection: IK, JP. Acquisition of cognitive data: FR, VA. Analysis of data: KB, MB, AL. Interpretation of data: KB, PA, JP, FR, VA. Drafting and writing of manuscript: KB, PA. Revision of manuscript: PA, JP, FR, VA, IK. All authors read and approved the final manuscript.

## Supplementary Material

Additional file 1 Construct validity according to the cognitive status defined from the attention_WMS score. Click here for file

Additional file 2 Construct validity according to the cognitive status defined from the PASAT score. Click here for file

Additional file 3 Associations between MusiQoL dimension scores and sociodemographic characteristics according to the cognitive status based on Wechsler Memory Scale. Click here for file

Additional file 4 Associations between MusiQoL dimension scores and sociodemographic characteristics according to the cognitive status based on PASAT. Click here for file
