# Supplementary material for: What is the relevance of quality of life assessment for patients with attention impairment?
Source: Health Qual Life Outcomes. 2013 Apr 25;11:70. doi: 10.1186/1477-7525-11-70 (PMC3640938; doi:10.1186/1477-7525-11-70)
Supplement: Additional file 1 — Construct validity according to the cognitive status defined from the attention_WMS score. [file 1477-7525-11-70-S1.docx]

**Additional file 1. Construct validity according to the cognitive status defined from the attention_WMS score**

|  | | Non-impaired N=75 | | | | | | | | | Impaired N=39 | | | | | | | | |
| --- | --- | --- | --- | --- | --- | --- | --- | --- | --- | --- | --- | --- | --- | --- | --- | --- | --- | --- | --- |
| Dom | Items | 1 | 2 | 3 | 4 | 5 | 6 | 7 | 8 | 9 | 1 | 2 | 3 | 4 | 5 | 6 | 7 | 8 | 9 |
| ADL | 1 | 0,62 |  |  |  |  |  | 0,60 |  |  | 0,57 |  |  |  | 0,71 |  |  |  |  |
|  | 2 |  |  |  |  |  |  | 0,76 |  |  | 0,57 |  | -0,45 |  |  |  |  |  |  |
|  | 3 | 0,57 |  |  |  |  |  | 0,51 |  |  | 0,64 |  |  |  | 0,45 |  |  |  |  |
|  | 4 | 0,79 |  |  |  |  |  |  |  |  |  |  |  |  | 0,86 |  |  |  |  |
|  | 5 |  |  |  |  |  |  | 0,80 |  |  | 0,38 |  |  |  | 0,73 |  |  |  |  |
|  | 6 | 0,66 |  |  |  |  |  |  |  |  |  |  | -0,69 |  |  |  |  |  |  |
|  | 7 | 0,75 | 0,40 |  |  |  |  |  |  |  | 0,75 |  |  |  |  |  |  |  |  |
|  | 8 | 0,66 | 0,53 |  |  |  |  |  |  |  | 0,74 |  |  |  |  |  |  |  |  |
| PWB | 9 |  |  |  |  | 0,83 |  |  |  |  |  | 0,79 |  |  |  |  |  |  |  |
|  | 10 |  |  |  |  | 0,78 |  |  |  |  |  | 0,90 |  |  |  |  |  |  |  |
|  | 11 |  |  |  |  | 0,31 | 0,49 |  |  |  |  | 0,84 |  |  |  |  |  |  |  |
|  | 12 |  |  |  |  | 0,65 | 0,41 |  |  |  |  | 0,65 |  |  |  |  |  |  |  |
| SPT | 13 |  | 0,69 |  |  |  |  |  |  |  |  |  |  |  |  |  |  |  | 0,86 |
|  | 14 |  | 0,87 |  |  |  |  |  |  |  |  |  |  |  |  |  |  |  | 0,85 |
|  | 15 |  | 0,57 |  |  |  |  |  |  |  | 0,60 |  |  |  |  |  |  |  |  |
|  | 16 |  | 0,63 |  |  |  |  |  |  |  | 0,61 |  |  |  |  |  |  |  |  |
| RFr | 17 |  |  | 0,85 |  |  |  |  |  |  |  |  |  |  |  |  |  | 0,76 |  |
|  | 18 |  |  | 0,83 |  |  |  |  |  |  |  |  |  |  |  |  |  | 0,91 |  |
|  | 19 |  |  | 0,88 |  |  |  |  |  |  |  |  |  |  |  |  |  | 0,69 |  |
| RFa | 20 |  |  |  | 0,57 | 0,52 |  |  |  |  |  |  | 0,68 | 0,43 |  |  |  |  |  |
|  | 21 |  |  |  | 0,67 |  |  |  |  |  |  |  | 0,82 |  |  |  |  |  |  |
|  | 22 |  |  |  | 0,85 |  |  |  |  |  |  |  | 0,76 |  |  |  |  |  |  |
| RHCS | 29 |  |  |  |  |  |  |  | 0,89 |  |  |  |  |  |  |  | 0,70 |  |  |
|  | 30 |  |  |  |  |  |  |  | 0,83 |  |  |  |  |  |  |  | 0,73 |  |  |
|  | 31 |  |  |  |  |  |  |  | 0,49 | -0,47 |  |  |  |  |  |  | 0,84 |  |  |
| SSL | 23 |  |  |  | 0,67 |  |  |  |  |  |  |  |  |  |  | 0,85 |  |  |  |
|  | 24 |  |  |  | 0,69 |  |  |  |  |  |  |  |  |  |  | 0,93 |  |  |  |
| COP | 25 |  |  |  |  |  |  |  |  | 0,53 |  |  |  | 0,79 |  |  |  |  |  |
|  | 26 |  |  |  |  |  |  |  |  | 0,85 |  |  |  | 0,77 |  |  |  |  |  |
| REJ | 28 |  |  |  |  |  | 0,89 |  |  |  |  | 0,43 |  | 0,57 |  | -0,49 |  |  |  |
|  | 29 |  |  |  |  |  | 0,84 |  |  |  |  | 0,57 |  |  |  | -0,56 |  |  |  |

ADL activity of daily living, PWB psychological well-being, RFr relationships with friends, SPT symptoms, RFa relationships with family, RHCS relationships with health care system, SSL sentimental and sexual life, COP coping, REJ rejection
