# Supplementary material for: What is the relevance of quality of life assessment for patients with attention impairment?
Source: Health Qual Life Outcomes. 2013 Apr 25;11:70. doi: 10.1186/1477-7525-11-70 (PMC3640938; doi:10.1186/1477-7525-11-70)
Supplement: Additional file 3 — Associations between MusiQoL dimension scores and sociodemographic characteristics according to the cognitive status based on Wechsler Memory Scale. [file 1477-7525-11-70-S3.docx]

**Additional file 3. Associations between MusiQoL dimension scores and sociodemographic characteristics according to the cognitive status based on Wechsler Memory Scale**

|  |  | Gender |  |  |  | Educational level | |  |  | Marital status | |  |  | Occupational status | |  |  |  |  |  |
| --- | --- | --- | --- | --- | --- | --- | --- | --- | --- | --- | --- | --- | --- | --- | --- | --- | --- | --- | --- | --- |
|  |  | Women | Men | p |  | Low | High | p |  | Single | Partnership | p |  | Not working | Working | p |  | Age° | EDSS° | MS duration° |
| ADL | NI | 31,8±23,3 | 33,5±20,2 | 0,437 |  | 32,0±19,8 | 32,8±22,7 | 0,975 |  | 30,9±21,3 | 33,9±22,4 | 0,578 |  | 25,6±16,8 | 42,7±23,7 | **0,003** |  | -0,024 | **-0,535**** | -0,030 |
|  | I | 27,7±18,7 | 30,1±16,3 | 0,488 |  | 26,2±17,7 | 32,1±18,2 | 0,310 |  | 30,5±19,0 | 26,3±17,0 | 0,549 |  | 27,0±18,5 | 34,4±16, 9 | 0,303 |  | -0,134 | -0,169 | -0,117 |
| PWB | NI | 46,9±25,9 | 60,1±21,2 | **0,024** |  | 48,8±26,2 | 54,3±24,2 | 0,445 |  | 51,8±26,7 | 53,7±23,2 | 0,585 |  | 54,3±21,6 | 54,9±27,8 | 0,919 |  | **0,254*** | 0,11 | 0,102 |
|  | I | 44,4±25,6 | 59,9±22,9 | 0,093 |  | 50,3±26,7 | 46,6±24,3 | 0,638 |  | 47,8±28,2 | 50,0±23,3 | 0,693 |  | 46,6±26,3 | 58,3±24,6 | 0,313 |  | -0,016 | 0,016 | -0,239 |
| RFr | NI | 63,3±23,6 | 59,1±27,1 | 0,554 |  | 57,0±27,7 | 63,0±24,3 | 0,429 |  | 56,0±29,2 | 65,7±20,9 | 0,194 |  | 64,7±24,7 | 61,2±25,1 | 0,555 |  | 0,123 | **0,279*** | 0,057 |
|  | I | 63,0±22,9 | 58,3±22,1 | 0,381 |  | 62,2±24,7 | 60,7±18,9 | 0,737 |  | 55,3±25,3 | 68,0±17,6 | 0,102 |  | 63,2±24,0 | 56,9±12,3 | 0,439 |  | -0,115 | 0,007 | 0,129 |
| SPT | NI | 55,2±24,9 | 64,0±19,4 | 0,074 |  | 45,7±24,5 | 63,9±20,5 | **0,004** |  | 56,6±23,1 | 61,1±22,8 | 0,438 |  | 58,6±23,7 | 60,9±22,5 | 0,685 |  | 0,054 | 0,064 | 0,002 |
|  | I | 48,6±22,4 | 50,0±20,7 | 0,884 |  | 47,7±19,1 | 51,3±26,2 | 0,726 |  | 45,7±23,8 | 52,3±19,5 | 0,277 |  | 46,3±20,9 | 46,9±17,6 | 0,843 |  | 0,164 | 0,119 | 0,022 |
| RFa | NI | 74,2±22,1 | 71,2±22,8 | 0,583 |  | 70,6±27,4 | 73,6±20,5 | 0,849 |  | 67,2±23,1 | 77,2±20,9 | 0,057 |  | 70,9±20,9 | 79,0±22,6 | 0,088 |  | 0,005 | 0,061 | -0,135 |
|  | I | 71,6±27,8 | 77,3±20,8 | 0,768 |  | 73,6±28,0 | 72,6±22,5 | 0,656 |  | 67,1±27,8 | 79,4±22,6 | 0,163 |  | 73,0±25,6 | 75,0±34,6 | 0,721 |  | -0,099 | 0,182 | -0,186 |
| RHCS | NI | 70,8±19,8 | 71,0±19,8 | 0,866 |  | 82,9±14,8 | 66,7±19,5 | **0,002** |  | 66,2±20,2 | 74,6±18,6 | 0,118 |  | 70,0±18,9 | 73,9±19,5 | 0,319 |  | -0,024 | -0,187 | 0,022 |
|  | I | 67,9±16,1 | 67,4±24,9 | 0,948 |  | 70,1±21,0 | 63,7±13,7 | 0,199 |  | 61,4±18,1 | 74,1±17,6 | 0,059 |  | 67,5±20,5 | 68,1±14,4 | 1,000 |  | 0,240 | 0,093 | -0,006 |
| SSL | NI | 49,7±30,3 | 41,7±32,9 | 0,382 |  | 41,9±33,6 | 47,5±30,9 | 0,630 |  | 31,3±31,9 | 55,6±27,6 | **0,003** |  | 41,8±32,1 | 53,6±33,8 | 0,173 |  | -0,007 | -0,092 | 0,011 |
|  | I | 50,46±34,4 | 43,8±30,8 | 0,500 |  | 52,6±35,7 | 41,4±27,7 | 0,343 |  | 43,8±35,7 | 53,3±30,9 | 0,382 |  | 51,8±33,3 | 50,0±30,6 | 0,837 |  | -0,253 | 0,008 | -0,185 |
| COP | NI | 56,3±28,0 | 59,5±30,1 | 0,495 |  | 57,2±29,9 | 57,9±28,8 | 0,949 |  | 55,5±33,0 | 59,5±25,4 | 0,753 |  | 60,5±30,0 | 58,7±25,1 | 0,668 |  | **0,310**** | 0,072 | -0,013 |
|  | I | 41,2±29,6 | 48,9±28,2 | 0,445 |  | 43,2±26,8 | 43,8±33,5 | 0,988 |  | 39,5±26,8 | 47,4±31,3 | 0,435 |  | 42,7±28,5 | 56,3±32,4 | 0,270 |  | -0,049 | -0,106 | 0,035 |
| REJ | NI | 60,9±32,7 | 71,2±33,3 | 0,113 |  | 73,7±30,9 | 62,7±33,7 | 0,184 |  | 56,6±36,2 | 72,6±29,1 | 0,067 |  | 68,0±32,9 | 64,7±31,2 | 0,587 |  | 0,195 | 0,158 | 0,174 |
|  | I | 60,65±38,1 | 81,8±24,6 | 0,122 |  | 63,0±37,7 | 73,2±32,5 | 0,532 |  | 71,1±35,1 | 62,5±36,8 | 0,382 |  | 65,5±36,1 | 77,1±30,0 | 0,484 |  | -0,112 | 0,056 | 0,058 |
| Index | NI | 56,4±11,4 | 59,2±15,1 | 0,374 |  | 55,4±12,5 | 58,5±13,4 | 0,437 |  | 51,5±14,2 | 61,7±10,8 | **0,005** |  | 57,7±11,5 | 60,26±16,1 | 0,415 |  | 0,231 | 0,096 | 0,142 |
|  | I | 52,8±11,2 | 58,2±14,87 | 0,231 |  | 54,3±12,6 | 54,2±12,2 | 0,703 |  | 51,4±13,2 | 57,0±11,1 | 0,133 |  | 53,8±12,7 | 58,1±11,0 | 0,442 |  | -0,140 | 0,047 | -0,129 |

ADL activity of daily living, PWB psychological well-being, RFr relationships with friends, SPT symptoms, RFa relationships with family, RHCS relationships with health care system, SSL sentimental and sexual life, COP coping, REJ rejection

NI non-impaired, I impaired

Bold values: p<0.05
