# Supplementary material for: What is the relevance of quality of life assessment for patients with attention impairment?
Source: Health Qual Life Outcomes. 2013 Apr 25;11:70. doi: 10.1186/1477-7525-11-70 (PMC3640938; doi:10.1186/1477-7525-11-70)
Supplement: Additional file 4 — Associations between MusiQoL dimension scores and sociodemographic characteristics according to the cognitive status based on PASAT. [file 1477-7525-11-70-S4.docx]

**Additional file 4. Associations between MusiQoL dimension scores and sociodemographic characteristics according to the cognitive status based on PASAT**

|  |  | Gender |  |  |  | Educational level | |  |  | Marital status | |  |  | Occupational status | |  |  |  |  |  |
| --- | --- | --- | --- | --- | --- | --- | --- | --- | --- | --- | --- | --- | --- | --- | --- | --- | --- | --- | --- | --- |
|  |  | Women | Men | p |  | Low | High | p |  | Single | Partnership | p |  | Not working | Working | p |  | Age° | EDSS° | MS duration |
| ADL | NI | 35,4±22,9 | 40,1±26,0 | 0,56 |  | 32,4±21,0 | 37,6±24,1 | 0,59 |  | 37,9±25,2 | 35,2±22,2 | 0,88 |  | 29,1±18,0 | 45,5±26,5 | 0,13 |  | -0,053 | -0,363 | 0,118 |
|  | I | 28,4±21,0 | 30,1±17,4 | 0,32 |  | 29,4±17,3 | 29,2±20,3 | 0,76 |  | 29,0±17,6 | 29,5±20,3 | 0,76 |  | 25,2±17,0 | 37,5±19,6 | **0,02** |  | -0,006 | **-0,359**** | 0,000 |
| PWB | NI | 49,5±26,5 | 57,3±14,5 | 0,46 |  | 44,5±26,8 | 53,1±24,1 | 0,51 |  | 54,8±28,5 | 48,4±22,0 | 0,60 |  | 47,9±22,6 | 53,4±27,9 | 0,55 |  | 0,134 | 0,128 | 0,002 |
|  | I | 41,3±26,6 | 58,9±21,8 | **0,01** |  | 47,6±26,0 | 53,0±25,2 | 0,40 |  | 48,1±26,5 | 52,9±24,8 | 0,35 |  | 52,1±24,4 | 55,9±26,4 | 0,64 |  | 0,129 | 0,099 | -0,039 |
| RFr | NI | 63,8±25,2 | 66,7±22,4 | 0,69 |  | 66,7±29,2 | 63,5±23,2 | 0,78 |  | 60,9±28,3 | 66,7±21,7 | 0,73 |  | 67,6±23,6 | 65,9±23,7 | 0,67 |  | -0,183 | -0,014 | -0,215 |
|  | I | 65,2±20,6 | 58,5±26,0 | 0,25 |  | 58,9±24,8 | 63,4±23,1 | 0,46 |  | 54,3±27,0 | 67,1±19,5 | **0,04** |  | 64,5±24,6 | 57,0±21,7 | 0,20 |  | **0,288*** | **0,368**** | **0,235*** |
| SPT | NI | 54,8±24,9 | 68,8±13,1 | 0,19 |  | 50,8±27,2 | 59,6±22,5 | 0,31 |  | 60,6±24,3 | 55,3±23,6 | 0,57 |  | 55,6±24,7 | 60,2±24,7 | 0,61 |  | 0,181 | 0,105 | -0,113 |
|  | I | 50,0±23,9 | 58,5±21,5 | 0,15 |  | 45,3±22,6 | 60,9±21,0 | **0,01** |  | 51,0±22,4 | 57,3±23,1 | 0,21 |  | 54,3±23,7 | 54,6±22,0 | 0,92 |  | 0,001 | 0,097 | 0,099 |
| RFa | NI | 73,7±23,7 | 83,3±18,3 | 0,36 |  | 75,0±31,8 | 75,7±19,8 | 0,69 |  | 80,1±19,1 | 72,4±25,0 | 0,36 |  | 73,6±21,4 | 84,1±16,9 | 0,17 |  | **-0,504**** | -0,057 | **-0,483**** |
|  | I | 71,0±27,2 | 71,4±22,5 | 0,83 |  | 67,0±28,4 | 74,0±21,5 | 0,41 |  | 58,3±26,6 | 80,9±17,8 | **0,00** |  | 69,7±24,7 | 74,5±27,7 | 0,36 |  | 0,160 | 0,055 | -0,025 |
| RHCS | NI | 72,8±18,9 | 72,2±16,4 | 0,69 |  | 88,5±14,0 | 67,4±16,5 | **0,00** |  | 67,9±20,4 | 75,9±16,4 | 0,47 |  | 69,4±20,2 | 75,8±15,1 | 0,49 |  | -0,157 | -0,290 | -0,225 |
|  | I | 69,7±16,6 | 66,7±22,7 | 0,73 |  | 73,3±20,0 | 64,5±19,6 | 0,06 |  | 65,9±20,0 | 69,7±20,2 | 0,47 |  | 68,8±19,3 | 68,9±21,7 | 0,78 |  | 0,136 | -0,074 | 0,119 |
| SSL | NI | 53,1±31,8 | 72,9±20,0 | 0,17 |  | 53,1±25,7 | 58,5±32,6 | 0,63 |  | 46,6±33,6 | 63,2±27,8 | 0,23 |  | 55,1±30,3 | 65,0±29,9 | 0,36 |  | -0,221 | -0,115 | 0,184 |
|  | I | 46,1±32,9 | 40,9±32,8 | 0,51 |  | 44,6±37,0 | 42,4±29,8 | 0,85 |  | 33,2±36,2 | 50,6±28,2 | **0,03** |  | 44,6±34,5 | 45,8±31,8 | 0,92 |  | 0,018 | 0,053 | -0,120 |
| COP | NI | 58,2±32,0 | 58,3±32,3 | 0,98 |  | 46,1±25,7 | 58,5±32,6 | 0,31 |  | 62,5±33,1 | 55,3±31,0 | 0,43 |  | 64,6±29,5 | 58,0±29,2 | 0,49 |  | 0,332 | 0,056 | 0,106 |
|  | I | 47,7±28,0 | 57,7±28,8 | 0,14 |  | 53,9±28,8 | 52,6±28,9 | 0,85 |  | 47,2±32,1 | 57,6±25,3 | 0,16 |  | 52,2±30,0 | 57,9±24,7 | 0,47 |  | 0,162 | 0,096 | -0,011 |
| REJ | NI | 59,6±30,5 | 68,8±35,1 | 0,46 |  | 60,9±27,1 | 61,5±32,7 | 0,88 |  | 56,7±30,5 | 64,5±31,8 | 0,45 |  | 61,1±30,9 | 62,5±28,5 | 0,91 |  | 0,008 | -0,153 | 0,108 |
|  | I | 64,8±35,8 | 71,8±32,3 | 0,39 |  | 71,6±33,0 | 66,6±34,7 | 0,41 |  | 66,1±36,3 | 70,4±32,3 | 0,65 |  | 72,8±32,3 | 68,4±32,6 | 0,56 |  | 0,179 | **0,243*** | 0,186 |
| Index | NI | 57,0±10,9 | 64,4±5,6 | 0,09 |  | 57,7±8,5 | 59,0±11,4 | 0,50 |  | 57,0±10,5 | 59,6±10,8 | 0,47 |  | 58,4±10,7 | 61,4±9,5 | 0,51 |  | -0,044 | -0,130 | 0,025 |
|  | I | 53,7±10,9 | 57,1±15,6 | 0,37 |  | 54,1±12,8 | 56,4±14,2 | 0,44 |  | 49,8±14,1 | 59,6±11,8 | **0,00** |  | 55,9±12,2 | 58,0±17,5 | 0,51 |  | 0,218 | 0,179 | 0,093 |

ADL activity of daily living, PWB psychological well-being, RFr relationships with friends, SPT symptoms, RFa relationships with family, RHCS relationships with health care system, SSL sentimental and sexual life, COP coping, REJ rejection

NI non-impaired, I impaired

Bold values: p<0,05
